# Profiles of physical frailty, social frailty, and cognitive impairment among older adults in rural areas of China: a latent profile analysis

**DOI:** 10.3389/fpubh.2024.1424791

**Published:** 2024-07-18

**Authors:** Qian Dong, Xiaolong Bu, Ting Wang, Man Liu, Feng Zhong, Cuiping Liu

**Affiliations:** ^1^School of Nursing, Shandong First Medical University & Shandong Academy of Medical Sciences, Taian, China; ^2^School of Public Health, Qingdao University, Qingdao, China

**Keywords:** older adults, physical frailty, social frailty, cognitive impairment, latent profiles analysis

## Abstract

**Background:**

As China rapidly ages, it has now become a deeply aging society with the largest number of older individuals in the world. The issue is particularly severe in rural areas. With the aging population growing and the older population expanding, health problems are becoming more prevalent among older individuals, particularly frailty and cognitive impairments. This study aimed to identify the profiles of physical frailty, social frailty, and cognitive impairment among older adults and explore the influencing factors.

**Methods:**

In this cross-sectional study, participants were recruited from six villages in four cities in Shandong Province, China from July to October 2023 through cluster random sampling. Latent profile analysis was used to determine the profiles of physical frailty, social frailty, and cognitive impairment. Chi-square tests and Mann–Whitney U tests were used for univariate analysis, while binary logistic regression was used to analyze the related factors.

**Results:**

Seven hundred and sixty-nine older adult care in rural areas showed two profiles: the “high cognitive function and low frailty” group (73.7%, *n* = 567) and the “low cognitive function and high frailty” group (26.3%, *n* = 202). A binary logistic regression found that older people were more likely to be aged 80 or older (OR = 2.253, *p* = 0.029), have a low income level (OR = 1.051, *p* = 0.007), have one or two (OR = 2.287, *p* = 0.004), or more than three chronic diseases (OR = 3.092, *p* = 0.002), and report moderate (OR = 3.406, *p* = 0.024) or poor health status (OR = 9.085, *p* < 0.001) in the “low cognitive function and high frailty” group. Meanwhile, older adults who have completed high school (OR = 0.428, *p* = 0.005) or junior college and above (OR = 0.208, *p* = 0.009), and engage in adequate physical activity (OR = 0.319, *p* < 0.001) were more likely to be in the “high cognitive function and low frailty” group.

**Conclusion:**

In the future, medical professors should increasingly prioritize promptly identifying and intervening in cognitive decline and frailty status in older individuals without delay.

## 1 Introduction

Population aging is a worldwide trend. Today, every country in the world is witnessing a swift growth in both the size and proportion of the population who are 60 years and older. Low- and middle-income countries are currently the most important observers and attestors experiencing the great change, such as China (1). The aging population in China is characterized by a shift in urban and rural areas, with rural areas aging at a higher level and at a faster rate than cities (2). The health problems of rural older adults are more serious than those of older individuals living in urban areas. Human aging is a complicated, individualized, and irreversible phenomenon that usually has an impact on physical, cognitive, and social abilities (3). With advancing age, older people are increasingly at risk of frailty and cognitive impairment (4).

Frailty is a multi-dimensional concept, including physiological, psychological, social and other areas (5, 6). It is an age-related condition, which drastically affects the quality of life and independence of older adults, as well as posing a tremendous burden on their families and society (7). Physical frailty is a vulnerability status characterized by a decline in physical reserve, reduced stress resistance, increased susceptibility of the body, and proneness to diseases (8). It is a severe consequence of the deterioration of multiple bodily functions (9), leading to fatigue, falls, extended sickness and even death (10). According to Fried frailty criteria, it is composed of five elements, including weight loss, exhaustion, low muscle strength, slow walking, low physical activity (11). Just like physical frailty, as individuals age, both their physical and psychological resilience diminishes, leaving them more susceptible to stress and illness, resulting in psychological distress and ultimately psychological frailty. Psychological frailty comprises four sets of components: mood problems, cognitive issues, other mental health concerns, and fatigue-related problems (12). Psychological frailty can be defined as a state of mental susceptibility and limited psychological resilience, combining cognitive, emotional, and fatigue-related factors (13). Even more importantly, social frailty is described as a state of deficiency in critical general and social resources, social behaviors, as well as self-management abilities essential for satisfying one’s social needs (14). In simpler terms, if people are unable to reach the crucial resources needed to meet their basic social needs, it indicates that the person is struggling with social frailty (15). Moreover, it has a detrimental effect on general well-being throughout all life stages, particularly during old age.

A prospective cohort study revealed that physical frailty affects the development of social frailty (16). In this study, the findings of 342 socially robust older adults living in the community at the two-year follow-up indicated that both gait speed and muscle strength were identified as crucial independent risk factors for future social decline. At the same time, research has shown that social frailty is a predictor of physical frailty (17). The results of a longitudinal study suggest that older adults who developed social frailty at baseline are at a higher risk of developing physical frailty. As a result, physical and social frailty may influence each other.

Cognitive impairment is another significant indicator of aging in the older population (18). Individuals’ cognitive function is the fundamental capacity to achieve and maintain a high-quality life (19). Frailty has a significant and negative influence on cognitive performance. Frailty has accelerated the deterioration of cognitive function in older individuals (20). Similarly, a prior study has demonstrated that older adults with subjective cognitive decline are more likely to be frail (21). At the present, an increasing number of studies have presented that frailty and cognitive function are interconnected, having a bidirectional relationship (22, 23).

Despite examining the characteristics of frailty or cognitive function from an individual perspective using latent class analysis in previous studies (24, 25), researchers have often considered one variable as the influencing factor and explored its relationship with another. Scholars seldom view these two potential conditions, which could coexist in older individuals, as a whole in order to examine their characteristics and relationships. As such, it is unclear what the current state of frailty and cognitive function is in older adults when viewed from an individual perspective.

In the current study, we chose physical frailty, social frailty, and cognitive impairment as the variables of interest and explored the heterogeneity of these variables among individuals based on all measurements through latent profile analysis. Additionally, certain variables with significant differences between profiles were incorporated into the multivariate analysis to determine the factors impacting the latent profiles.

## 2 Methods

### 2.1 Design and participants

The study employed a cross-sectional design, and participants were recruited from six villages in four cities in Shandong Province, China from July to October 2023 through cluster random sampling. Participants were eligible if they were (1) aged 60 years or older, (2) living in rural areas, and (3) able to understand and cooperate with the study. They were excluded if they (1) had hearing or visual impairments, (2) had multiple physical or psychological illnesses, or (3) refused to answer or provide incomplete responses to the questionnaire.

### 2.2 Sample size

It is generally suggested that the sample size for multivariate statistics should be more than 10 events per variable (26). In our study, the regression analysis included 16 observational variables, so the sample size should be a minimum of 160 people. The final sample included 769 older adults living in communities.

### 2.3 Measurements

Socio demographic characteristics included age, sex, BMI, education level, income level, marital status, number of children, frequency of visits by family and friends, smoking, drinking, number of chronic diseases, self-reported health status, and the use of walking aids.

#### 2.3.1 International physical activities questionnaire

The International Physical Activity Questionnaire (IPAQ) is a reliable tool used to measure physical activity in many countries. It has shown good reliability and validity (27). The Chinese version of the International Physical Activity Questionnaire short form (IPAQ-C) consists of seven questions, covering four activities: vigorous intensity activities, moderate intensity activities, walking, and sitting (28). They were assigned 8.0, 4.0, 3.3, and 1.1 metabolic equivalent (MET), respectively. Total physical activity is shown as metabolic equivalent (MET) minutes per day. The total metabolic equivalent/min (MET-min) was calculated using the formula: (8.0 × vigorous-intensity activity minutes × days) + (4.0 × moderate-intensity activity minutes × days) + (3.3 × walking minutes ×days) + (1.1 × sitting minutes × days). The physical activity levels were divided into three categories: low (<600 MET-min/week), medium (600–2,999 MET-min/week), and high (≥3,000 MET-min/week).

#### 2.3.2 Mini nutritional assessment short form

The Mini Nutritional Assessment (MNA) is an efficient tool to assess the nutritional status of older adults, and it can be completed in about 10 min (29). MNA-SF comprises six questions chosen from MNA, covering weight loss, BMI, eating problems, mobility limitations, acute illnesses, and neuropsychological issues (30). The MNA-SF total score is 14, with scores of <8 indicating malnutrition, scores of 8–11 indicating a risk of malnutrition, and scores of >11 indicating no malnutrition. The Cronbach’s α was 0.80.

#### 2.3.3 Mini-mental state examination

MMSE is a universal questionnaire used to assess cognitive impairment, comprising 5 cognitive domains: orientation in time and place, memory, attention and calculation, recall, and language. It includes 30 questions with a maximum score of 30, where higher scores suggest superior cognitive abilities (31). MMSE-C is a specific cognitive evaluation tool that has been developed based on China’s realities to evaluate the cognitive condition of the Chinese older population (32). The Cronbach’s α was 0.83, and the reliability and validity were good.

#### 2.3.4 Fried frailty phenotype

Frailty was measured with the Fried’s frailty phenotype (33). It consists of five criteria: unintentional weight loss 10 kg during last year, lack of energy and fatigue, low handgrip strength, low gait speed, low physical activity. If an individual meets any one of the five criteria, then the score will be 1; otherwise, the score will be 0. The total scores range from 0 to 5, and higher scores indicate more severe frailty. Participants scored 0 were defined as non-frail, scored 1 or 2 as prefrail, and scored ≥ 3 as frail. The Chinese Fried frailty phenotype showed good reliability and validity, with a Cronbach’s α of 0.93 (34).

#### 2.3.5 Social frailty scale

Social frailty was assessed using an 8-item Social Frailty Scale (SFS-8) (35), which includes 8 items in three dimensions: social resources (three items), social activities and financial resources (three items), and social need fulfillment (two items). The total score of the scale ranged from 0 to 8, with larger scores indicating higher levels of social frailty. Participants with a score of 0–1 was considered non-SF; 2–3 was considered pre-SF; and a score of ≥4 indicated SF.

### 2.4 Data collection

Before data collection, our research team members all received uniform training to maintain consistency and standardization in this task. We gather data by distributing questionnaires face-to-face and then collecting them. While collecting data, team members explained the study to eligible participants and assisted those who had difficulty in completing the questionnaire.

### 2.5 Data analysis

In this study, SPSS 26.0 was conducted to statistical description and analysis, while Mplus 8.3 was used for latent profile analysis (LPA). Firstly, categorical variables were presented as frequencies and percentages, while non-normal continuous variables were shown as the median (M) and interquartile range (IQR). Secondly, the correlations of study variables in Spearman’s product moment were examined. Thirdly, we determined the optimal model by progressively increasing the number of profiles in the model and comparing the fitness. In order to determine the appropriate number of profiles, we evaluated several metrics, including the Akaike Information Criterion (AIC), the Bayesian Information Criterion (BIC), the Sample-Size-Adjusted BIC (aBIC), the Entropy, the Lo–Mendell–Rubin Likelihood Ratio Test (LMR), and the Bootstrap Likelihood Ratio Test (BLRT). A smaller value of the first three classes indicated a better fit. Entropy was used to assess classification accuracy, with a greater value indicating better accuracy. When the value surpasses 0.8, the accuracy will exceed 90% (36). The LMR and BLRT were used to compare the current model with the previous one, and if the probability value is significant (*p* < 0.05), it indicates that a k-profile model is better than a k-1 profile model (37). Thirdly, we performed a χ^2^ test or Fisher exact test and a Mann–Whitney U test to compare the characteristics of subgroups within the population and make inter-group comparisons. The variables with statistical significance in univariate analysis were included in a multivariate analysis to identify the factors that influenced the latent profiles. A *p*-value of <0.05 indicated statistical significance.

## 3 Results

### 3.1 Participants’ characteristics

Table 1 presents the characteristics of the participants. The participants ranged in age from 60 to 93 years old (72.17 ± 5.77), with the majority falling between 70 and 79 years old. The majority of older people were married (82.8%, *n* = 637). Over half of them had 3 to 5 children (54.6%, *n* = 420). Despite the fact that over three-fifths of the participants do not smoke (63.5%, *n* = 488) or drink (69.7%, *n* = 536), most still consider their physical health to be moderate (73.2%, *n* = 563).

**Table 1:** Characteristics of participants (*N* = 769).

Characteristics	*N* (*M*)	% (IQR)
**Age**		
60–69	279	36.3
70–79	409	53.2
≥80	81	10.5
**Sex**		
Male	391	50.8
Female	378	49.2
**BMI**		
<18.5	39	5.1
18.5 ~ 23.9	346	45.0
24.0 ~ 27.9	256	33.3
≥28.0	128	16.6
**Education level**		
Primary school and below	334	44.7
Junior school	242	31.5
High school	136	17.7
Junior college or above	47	6.1
**Income level**		
Good	273	35.5
Moderate	440	57.2
Bad	56	7.3
**Marital status**		
Married	637	82.8
Unmarried/Divorced/Widowed	132	17.2
**Co-residence**		
With spouse	600	78
With children	76	9.9
Alone	93	12.1
**Number of children**		
≤2	319	41.5
3–5	420	54.6
>5	30	3.9
**Frequency of visits by family and friends**		
Usually	133	17.3
Occasionally	600	78.0
Hardly ever	36	4.7
**Smoking**		
No	488	63.5
Yes	281	36.5
**Drinking**		
No	536	69.7
Yes	233	30.3
**Number of chronic diseases**		
0	200	26.0
1–2	464	60.3
≥3	105	13.7
**Self-reported health status**		
Good	108	14.0
Moderate	563	73.2
Bad	98	12.7
**Use of walking aids**		
No	717	93.2
Yes	52	6.8
**Physical activity**		
Low	44	5.7
Medium	205	26.7
High	520	67.6
**Nutritional status**		
Malnutrition	4	0.5
A risk of malnutrition	288	37.5
No malnutrition	477	62.0

### 3.2 Correlations, median, and interquartile range for the study variables

The correlations, medians, and interquartile ranges for the study variables are presented in Table 2, indicating significant associations among all of the variables.

**Table 2:** Spearman’s product moment correlation coefficients of study variables.

Variables	Cognitive function	Physical frailty	Social frailty	*M*	IQR
Cognitive function	–			22	7
Physical frailty	−0.308**	–		1	2
Social frailty	−0.114**	0.189**	–	1	2

**p < 0.01.

### 3.3 Results of latent profile analysis

In Table 3, as the number of profiles increases from one to four, there is a gradual decrease in AIC, BIC and aBIC, with a consistent *p*-value of BLRT <0.05, as well as an increase in entropy. However, some proportions in the three-profile model account for too few people, and the *p*-value of LMRT is >0.05 in both the three- and four-profile models. Considering the model performance, practical significance, and interpretability, the final optimal model determined was the two-profile model. As shown in Table 4, the average attribution probability of community older adults belonging to the profile ranged from 83.3 to 93.8%, indicating the reliability of the LPA results in this study.

**Table 3:** Latent profile analysis for model fit statistics.

Profile	k	Likelihood	AIC	BIC	aBIC	LMRT (*P*)	BLRT (*P*)	Entropy	Proportion
1	6	−3271.990	6555.980	6583.851	6564.798				
2	10	−3189.639	6399.273	6445.724	6413.969	0.0000	0.0000	0.707	0.737/0.263
3	14	−3164.632	6357.263	6422.295	6377.838	0.1534	0.0000	0.798	0.672/0.309/0.018
4	18	−2613.336	5262.673	5346.285	5289.126	0.2143	0.0000	1.000	0.397/0.256/0.124/0.224

k, free parameters; AIC, Akaike information criterion; BIC, Bayesian information criterion; aBIC, adjusted BIC; LMRT, Lo–Mendell-Rub test; BLRT, Bootstrap Likelihood ratio test.

**Table 4:** Average attribution probabilities for each latent profile.

Class	Profile 1	Profile 2
Profile 1	0.938	0.062
Profile 2	0.167	0.833

### 3.4 Naming of latent profile

**Figure 1:**
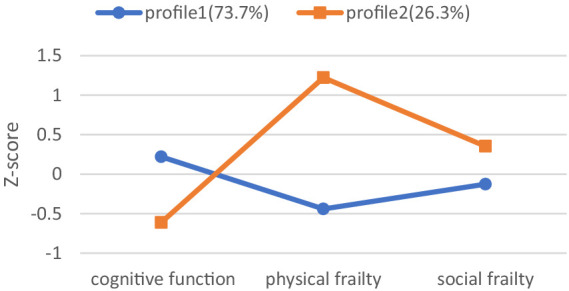
Two-profile model and probability on study variables in z-score format.

As we can see, the latent profiles had different characteristics regarding the study variables in Figure 1. Five hundred and sixty-seven community-dwelling older adults (73.7%) had higher scores for cognitive function, lower scores for physical frailty, and social frailty in profile 1, which was labeled as the “high cognitive function and low frailty” group. Two hundred and two community-dwelling older adults (26.3%) had lower scores for cognitive function, higher scores for physical frailty, and social frailty in profile 2, which was classified as the “low cognitive function and high frailty” group.

### 3.5 Inter-profile characteristic differences

The differences in demographic characteristics, cognitive function, physical frailty, and social frailty between two subgroups were compared using the chi-square test, Fisher exact test, and Mann–Whitney U test, as shown in Table 5. The results indicated significant differences in age, education level, income level, marital status, co-residence, number of children, drinking, number of chronic diseases, self-reported health status, use of walking aids, physical activity, nutritional status, cognitive function, physical frailty, and social frailty (*p* < 0.05). In both subgroups, the majority of community-dwelling older adults were aged 70–79 years old, had a primary school education or lower, held a middle-income status, and were married and living with a spouse. It is interesting to observe that the “high cognitive function and low frailty” group has a greater number of older adults with high physical activity and good nutritional status.

**Table 5:** Inter-profile characteristic differences.

Characteristics	High cognitive function and low frailty *n* = 567 (73.7%) *n* (%) or M (IQR)	Low cognitive function and high frailty *n* = 202 (26.3%) *n* (%) or M (IQR)	*Χ*^2^/*Z*	*p*
Age			41.698	<0.001
60–69	233 (41.1)	46 (22.8)		
70–79	295 (52.0)	114 (56.4)		
≥80	39 (6.9)	42 (20.8)		
Sex			0.013	0.908
Male	289 (51.0)	102 (50.5)		
Female	278 (49.0)	100 (49.5)		
BMI			0.230	0.973
<18.5	30 (5.3)	9 (4.5)		
18.5 ~ 23.9	254 (44.8)	92 (45.5)		
24.0 ~ 27.9	189 (33.3)	67 (33.2)		
≥28.0	94 (16.6)	34 (16.8)		
Education level			22.805	<0.001
Primary school and below	228 (40.2)	116 (57.4)		
Junior school	185 (32.6)	57 (28.2)		
High school	111 (19.6)	25 (12.4)		
Junior college or above	43 (7.6)	4 (2.0)		
Income level			29.415	<0.001
Good	224 (39.5)	49 (24.3)		
Moderate	316 (55.7)	124 (61.4)		
Bad	27 (4.8)	29 (14.4)		
Marital status			12.587	<0.001
Married	486 (85.7)	151 (74.8)		
Unmarried/Divorced/Widowed	81 (14.3)	51 (25.2)		
Co-residence			16.701	<0.001
With spouse	463 (81.7)	137 (67.8)		
With children	46 (8.1)	30 (14.9)		
Alone	58 (10.2)	35 (17.3)		
Number of children			8.516	0.014
≤2	249 (43.9)	70 (34.7)		
3–5	301 (53.1)	119 (58.9)		
>5	17 (3.0)	13 (6.4)		
Frequency of visits by family and friends			1.845	0.397
Usually	92 (16.2)	41 (20.3)		
Occasionally	449 (79.2)	151 (74.8)		
Hardly ever	26 (4.6)	10 (5.0)		
Smoking			3.385	0.066
No	349 (61.6)	139 (68.8)		
Yes	218 (38.4)	63 (31.2)		
Drinking			5.543	0.019
No	382 (67.4)	154 (76.2)		
Yes	185 (32.6)	48 (23.8)		
Number of chronic diseases			37.185	<0.001
0	178 (31.4)	22 (10.9)		
1–2	326 (57.5)	138 (68.3)		
≥3	63 (11.1)	42 (20.8)		
Self-reported health status			83.764	<0.001
Good	45 (7.9)	63 (31.2)		
Moderate	428 (75.5)	135 (66.8)		
Bad	94 (16.6)	4 (2.0)		
Use of walking aids			44.061	<0.001
No	549 (96.8)	168 (83.2)		
Yes	18 (3.2)	34 (16.8)		
Physical activity			115.101	<0.001
Low	8 (1.4)	36 (17.8)		
Medium	124 (21.9)	81 (40.1)		
High	435 (76.7)	85 (42.1)		
Nutritional status			22.305	<0.001^a^
Malnutrition	0 (0.0)	4 (2.0)		
A risk of malnutrition	192 (33.9)	96 (47.5)		
No malnutrition	375 (66.1)	102 (50.5)		
MMSE	23 (6)	19 (5)	−11.750	<0.001
FFP	1 (1)	2 (1)	−20.725	<0.001
SFS	1 (2)	2 (2)	−6.213	<0.001

### 3.6 Influences of factors on the latent profiles

A binary logistic regression was conducted to explore the factors influencing the two subgroups based on the LPA results. Table 6 indicates that age, education level, income level, number of chronic diseases, self-reported health status, and physical activity have statistically significant influences on the latent profiles. When comparing the “high cognitive function and low frailty” group with the “low cognitive function and high frailty” group, it was found that older individuals in the latter group were more likely to be aged 80 or older (OR = 2.253, *p* = 0.029), have a low income level (OR = 1.051, *p* = 0.007), have one or two (OR = 2.287, *p* = 0.004), or even more than three chronic diseases (OR = 3.092, *p* = 0.002), and report moderate (OR = 3.406, *p* = 0.024) or poor health status (OR = 9.085, *p* < 0.001). Meanwhile, older adults in the former group were found to have completed high school (OR = 0.428, *p* = 0.005) or junior college and above (OR = 0.208, *p* = 0.009), and engage in adequate physical activity (OR = 0.319, *p* < 0.001).

**Table 6:** Binary logistic regression for the latent profiles.

Characteristics	*B*	SE	Wald χ^2^	Exp(B)	95%	*p*
**Age**						
60–69			5.678			0.058
70–79	0.448	0.234	3.672	1.565	0.990, 2.473	0.055
≥80	0.812	0.372	4.775	2.253	1.087, 4.669	0.029
**Education level**						
Primary school and below			13.624			0.003
Junior school	−0.450	0.234	3.706	0.637	0.403, 1.008	0.054
High school	−0.849	0.306	7.714	0.428	0.235, 0.779	0.005
Junior college or above	−1.571	0.599	6.887	0.208	0.064, 0.672	0.009
**Income level**						
Good			7.619			0.022
Moderate	0.357	0.228	2.453	1.429	0.914, 2.233	0.117
Bad	1.051	0.387	7.364	2.861	1.339, 6.112	0.007
**Marital status**						
Unmarried/Divorced/Widowed	−0.786	−0.587	1.794	0.456	0.144, 1.439	0.180
**Co-residence**						
With spouse			3.249			0.197
With children	0.508	0.438	1.345	1.663	0.704, 3.925	0.246
Alone	1.161	0.645	3.243	3.193	0.903, 11.299	0.072
**Number of children**						
≤2			2.344			0.310
3–5	0.138	0.215	0.410	1.148	0.753, 1.750	0.522
>5	−0.552	0.480	1.320	0.576	0.225, 1.476	0.251
**Drinking**						
Yes	−0.263	0.235	1.248	0.769	0.485, 1.219	0.264
**Number of chronic diseases**						
0			11.029			0.004
1–2	0.827	0.284	8.486	2.287	1.311, 3.991	0.004
≥3	1.129	0.359	9.889	3.092	1.530, 6.248	0.002
**Self-reported health status**						
Good			20.308			<0.001
Moderate	1.226	0.542	5.105	3.406	1.176, 9.863	0.024
Bad	2.207	0.587	14.143	9.085	2.877, 28.694	<0.001
**Use of walking aids**						
Yes	0.701	0.410	3.008	2.035	0.912, 4.540	0.083
Physical activity	−1.143	0.172	43.896	0.319	0.227, 0.447	<0.001
Nutritional status	−0.333	0.196	2.890	0.717	0.488, 1.052	0.089

## 4 Discussion

The main purpose of this study was to classify subgroups of cognitive function, physical frailty, and social frailty in community-dwelling older individuals. The results of LPA determined two subgroups - the “high cognitive function and low frailty” group and the “low cognitive function and high frailty” group.

Specifically, the older individuals belonging to the “high cognitive function and low frailty” group, accounting for 73.7% of the total, were identified by their better cognitive function and fewer frailties. The older community members in the “low cognitive function and high frailty” group, comprising 26.3% of the total, were identified by their poorer cognitive function and more severe frailties. Levels of physical frailty and social frailty were found to be similar, as they were either high or low simultaneously in two latent profiles. Moreover, both were found to have a negative association with cognitive function. This result supported our classification of subgroups for cognitive function, physical frailty, and social frailty in older individuals living in the community. The findings of this research were consistent with a prior systematic review, which indicated that cognitive decline and physical frailty frequently co-occur among older individuals (38). In addition, two studies conducted in Japan have also shown that social frailty is associated with both cognitive impairment and physical frailty in older individuals living in the community, with these symptoms often overlapping (39, 40). A possible explanation for this phenomenon was that as older adults experience the debilitating syndrome, they may have low physical activity, slow movement, fatigue, and weakness. Consequently, they may avoid social activities, participate less in social activities, shrink their social circle, and ultimately develop social frailty. Conversely, social frailty can lead to a decrease in social activities for older adults, smaller social circles, reduced motor function, declining cognitive abilities, and ultimately physical and cognitive deterioration.

Our research also aimed to explore the factors that influence the classification of cognitive function and frailty in older members of the community. Through our study, we have identified age, education level, income level, number of chronic diseases, self-reported health status, and physical activity as significant influencers of cognitive performance, physical frailty, and social frailty in older adults living in the community.

Age was identified as a risk factor for cognitive function and frailty status in community-dwelling older adults in this study. Frailty, a prevalent age-related geriatric syndrome, frequently accompanies cognitive decline in older individuals (41). The coexistence of physical frailty and cognitive decline in older people is defined as cognitive frailty (42). As individuals age, their physiological functions tend to deteriorate, causing a reduction in their visual, auditory, and perceptual capacities, a decline in their physical performance, and low levels of physical activity, ultimately leading to physical frailty (9). Moreover, a previous study conducted on older adults in Shanghai also found that advanced age (81–85 years old) is associated with an increased risk of suffering from both physical frailty and cognitive impairment concurrently (43). Conversely, with a decline in physical and cognitive abilities, older individuals tend to self-isolate, which reduces their social engagement, interaction, and perceived social support, ultimately leading to social frailty.

Educational level and financial status as influencing factors of cognitive function and frailty status of older individuals in the community has been confirmed in this study. In rural areas, older adults often have access to fewer educational opportunities and resources, resulting in a lower level of education than their urban peers (44). An analysis using data from the Birjand Longitudinal Aging Study (BLAS) found that the level of education has an impact on physical, cognitive, psychological, and social frailty, as well as the relationships between them, among community-dwelling older adults (45). A lower income level is also a significant risk factor. Older individuals in rural areas predominantly depend on income from agricultural labor and odd jobs, lacking a stable source of income, which ultimately results in lower overall income levels compared to urban older adults (46). A systematic review of longitudinal studies has revealed that a lower income level has been identified as a risk factor associated with the development or progression of frailty in older adults living in the community (47). Social frailty among older individuals is also influenced by their educational level (48). It is our speculation that having sufficient financial resources guarantees a good quality of life for older adults in their later years, delaying the onset of frailty. Meanwhile, having enough wealth reserves can also help maintain the crucial social connections of older adults, increase their involvement in social activities, and slow the progression of social decline. If the physical and social functions of older adults remain normal, their cognitive function is also usually not impacted.

Number of chronic diseases and self-reported health status were significant in this study. With advancing age, individuals become more prone to weakness and illness, making them more susceptible to chronic diseases and frailty. The onset of chronic diseases can deteriorate physical function, diminish resistance to external stimuli, and ultimately increase the risk of frailty (49). Chronic illnesses and frailty are closely related conditions that often worsen each other and have a significant negative impact on the health and quality of life of older individuals (50). Moreover, a study revealed that community-dwelling older individuals with multiple chronic diseases have a higher level of frailty (51). More and more concrete evidence indicates that chronic diseases, particularly the presence of multiple chronic diseases, are significant predictors of poor self-rated health (52, 53). An individual’s self-reported health status is a subjective perception of their overall physical and mental well-being. Because chronic diseases are long-lasting and cannot be cured, older individuals often experience poor self-health status, negative emotions, and low life satisfaction (54). As a result, we speculated that they may refuse to participate in social activities, be more prone to depressive symptoms, and experience social frailty. A prior study found that older adults living alone with poor self-rated health are more likely to be depressed (55), which supports our findings in this study.

Older individuals with low levels of physical activity are at a higher risk of developing frailty compared to those with high levels of physical activity. Regular exercise was shown in a previous study to have a significant negative correlation with prefrailty and frailty (56). Physical exercise can enhance muscle strength in older individuals (57), diminish age-related inflammatory responses (58), enhance bodily functions, and thus delay and lessen frailty (59). As more research emerges, it is becoming increasingly clear that exercise has the potential to improve cognitive function in older adults by activating individual physiological mechanism (60) and providing psychological benefits (61). Additionally, engaging in physical activity can reduce social frailty, alleviating feelings of loneliness through interaction with others and building new social connections through participation in community activities (62).

## 5 Limitations

This study has some limitations. Firstly, since this study is cross-sectional, the results should not be interpreted as causal. In order to confirm the causal relationship, a longitudinal study should be conducted in the future. Secondly, certain sociodemographic variables and social frailty are self-reported, which may cause subjective bias. In future studies, further scientific measurements should be used. Thirdly, due to study constraints, variables such as sleep quality, healthy habits, and other factors that may affect cognitive function and frailty status in older individuals were not accounted for or included in the regression analysis. In future studies, we can gather more related variables in order to conduct a more comprehensive and thorough analysis of the influencing factors.

## 6 Conclusion

Our study divided cognitive function and frailty status in older adults into two subgroups: the “high cognitive function and low frailty” group, and the “low cognitive function and high frailty” group, each with distinct group characteristics. It indicates that frailty and cognitive impairment often coexist in older individuals, and they reciprocally impact each other. Older adults with cognitive impairments are more susceptible to physical and social decline, and vice versa. Age, education level, income level, number of chronic diseases, self-reported health status, and physical activity were found to be influencing factors for cognitive function and frailty status in older people. This finding improves our understanding of cognitive function and frailty status in older adults and implies that we should identify and intervene in cognitive decline and frailty status in older individuals in a multidimensional and comprehensive approach as soon as possible.

## Data availability statement

The raw data supporting the conclusions of this article will be made available by the authors, without undue reservation.

## Ethics statement

The studies involving humans were approved by the Ethics Committee of Qingdao University (QDU310 HEC-2022278). The studies were conducted in accordance with the local legislation and institutional requirements. Written informed consent was provided by the participants themselves or their legal guardians/next of kin.

## Author contributions

QD: Conceptualization, Data curation, Writing – original draft. XB: Data curation, Writing – original draft. TW: Investigation, Software, Writing – original draft. ML: Investigation, Software, Writing – review & editing. FZ: Writing – review & editing, Project administration, Supervision. CL: Writing – review & editing, Funding acquisition, Project administration.
